# Cataloguing the bacterial diversity in the active ectomycorrhizal zone of *Astraeus* from a dry deciduous forest of Shorea

**DOI:** 10.3897/BDJ.9.e63086

**Published:** 2021-05-19

**Authors:** Vineet Vishal, Somnath Singh Munda, Geetanjali Singh, Shalini Lal

**Affiliations:** 1 Department of Botany, Dr Shyama Prasad Mukherjee University, Ranchi-834008, India Department of Botany, Dr Shyama Prasad Mukherjee University Ranchi-834008 India; 2 Department of Botany, Bangabasi Evening College, Kolkata-700009, India Department of Botany, Bangabasi Evening College Kolkata-700009 India

**Keywords:** *
Astraeus
*, Ectomycorrhizae, Gammaproteobacteria, microbiome, PSB, OTUs

## Abstract

The plant microbiome has been considered one of the most researched areas of microbial biodiversity, yet very little information is available on the microbial communities prevailing in the mushroom's ectomycorrhizosphere. Ectomycorrhizal symbioses often result in the formation of a favourable niche which enables the thriving of various microbial symbionts where these symbionts endorse functions, such as quorum sensing, biofilm formation, volatile microbial compound (VOC) production, regulation of microbial gene expression, symbiosis and virulence. The identification of hidden uncultured microbial communities around the active ectomycorrhizal zone of *Astraeus* from dry deciduous sal forest of Jharkhand, India was carried out using MinION Oxford Nanopore sequencing of 16S rRNA amplicons genes. High richness of Operational Taxonomic Units (1,905 OTUs) was observed. We recorded 25 distinct phyla. Proteobacteria (36%) was the most abundant phylum, followed by Firmicutes (28%), Actinobacteria (10%) and Bacteroidetes (6%), whereas Gammaproteobacteria was the most abundant class of bacterial communities in the active ectomycorrhizal zone. The ectomycorrhizosphere soil has abundant phosphate-solubilising bacteria (PSB). This is the first report of the ectomycorrhizosphere microbiome associated with *Astraeus*.

## Introduction

Mycorrhizal symbioses are ubiquitous and form a major component of the microbiota in most boreal, temperate and dry deciduous tropical forest ecosystems. They contribute huge amounts of organic carbon fluxes from leaf-litter, wood or plant biomass, resulting in an enrichment of the microbial populace and their associated functions. The ectomycorrhizosphere is a zone of active interchange between plant root and soil microorganisms (ectomycorrhizal fungi and bacteria) that can inhibit or stimulate each other (Poole et al. 2001). The ectomycorrhizosphere also serves as a nutritional hotspot for microbes which benefit forest trees in a number of ways. Moreover, the ectomycorrhizosphere microbiome is driving processes like quorum sensing, regulation of microbial gene expression, symbiosis, biofilm formation, antibiotic production, motility, conjugation, virulence etc. (Churchland and Grayston 2014). Garbaye (1994) and Bonfante and Anca (2009) suggested that bacteria could potentially stimulate mycorrhizal formation, assist in the fungal-plant recognition system and receptivity of the host root to the mycorrhizal fungus. Uroz et al. (2007), Uroz and Oger (2015) isolated 61 bacterial strains from the ectomycorrhizosphere of an oak forest, where they have experimentally shown that bacteria (*Burkholderia*, *Collimonas*, *Pseudomonas* and *Sphingomonas*) and ectomycorrhizal fungi (*Scleroderma citrinum*) are jointly involved in mineral weathering and solubilisation processes.

Mushrooms are one of the finest creations in nature and exhibit wide variation. The wild edible mushrooms of *Astraeus* Phosri. (order Boletales), inhabiting the roots of *Shorea robusta* Gaertn., are a group of epigeous non-hygroscopic macro fungi found only during the monsoon season. They are recognised by the star-like pattern at maturity (Phosri et al. 2007) and are commonly known as earthstar. These ectomycorrhizal edible fungi grow extensively in the sandy and red laterite soil of Dipterocarp sal dry deciduous forest. Owing to their high nutritional property, these mushrooms are sold regularly in local markets during the monsoon season. However, due to changes in climatic conditions, decrease in rainfall, global warming and unauthorised anthropogenic influence, the production is declining rapidly, causing serious threats to these mushrooms. Although there are many reports on the morphology, photochemistry and nutritional property of *Astraeus*, little is known about the effect of bacteria on the growth and development of mushrooms of *Astraeus*. This may be one of the reasons that, despite nearly a decade of research, attempts to cultivate *Astraeus* have largely been unsuccessful (Trappe 1967, Petcharat 2003, Biswas et al. 2017, Biswas et al. 2011).

A detailed study on the biology and fruiting body production of *Astraeus* in a forest of sal and the co-occurrence between this fungus and other microbes (especially bacteria), is very important for a successful cultivation of mushroom and for a better yield. The main objective of the present work was to collect soil samples from a dry deciduous sal forest and to analyse the abundance of microorganisms around the ectomycorrhizosphere of *Astraeus*. In order to evaluate the optimum microbial content for proper nourishment of the taxon, the MinION Oxford Nanopore 16s amplicon sequencing platform was used.

## Material and Methods

### Collection and sample preparation

**Figure 1.**
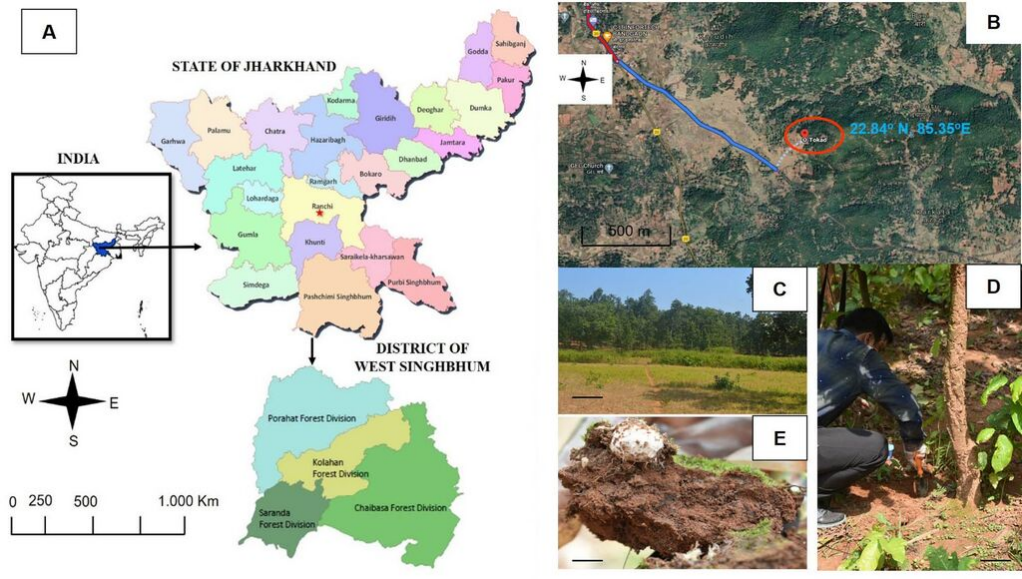
Area of study and collection of soil samples. **A.** Location and collection sites of soil samples from Porahat forest division (22.84°N, 85.35°E) of West Singhbhum District of Jharkhand State of India; **B.** Satellite image of the collection site; **C.** Natural vegetation of forest of Shorea from Porahat forest division; **D**-**E.** Collection of ectomycorrhizosphere soil samples. Scale bar C-D = 20 mm.

Rhizosphere samples of ectomycorrhizosphere (RUGA-1) were collected from the village of Bandgaon under the Porahat forest division, West Singhbhum, Jharkhand, India (22.84°N, 85.35°E; Fig. 1) from a dry deciduous forest of Shorea during the monsoon season in July 2019. The soil in this region is red laterite and sandy, having a thin organic layer. The soil samples (n = 3) were collected at a depth of 5–10 cm. The separation of the soil samples into ectomycorrhizosphere were performed in the lab. Plant roots were carefully eliminated from the soil and shaken gently to remove loosely adhering soil. Ectomycorrhizosphere soils were sieved (2 mm mesh) and homogenised prior to freezing (Uroz et al. 2010).

### DNA extraction, 16S rRNA gene amplification and sequencing

From a minimum of 1 g of soil, DNA was isolated using the EXpure Microbial DNA isolation kit (BogarBio Bee stores Pvt Ltd). DNA concentration was measured using a Qubit Flurometer 3.0 and DNA was stored at -20°C. Full-length 16S rRNA gene was amplified using the primers 27F (5'-AGAGTTTGATCCTGGCTCAG-3') and 1492R (5'-GGTTACCTTGTTACGACTT-3'). Metagenomic 16s amplicon sequencing was performed by taking 1 µg of DNA template using MinION Oxford Nanopore platform at Yaaz Xenomics (Coimbatore, India). The raw fastq files were uploaded to the metagenome rapid annotation using subsystem technology (MG-RAST server; see Aziz et al. 2008) and annotated using default parameters. Artificial duplicate reads were eliminated using DRISEE (Duplicate Read Inferred Sequencing Error Estimation; see Gomez-Alvarez et al. 2009). Taxonomic assignment of the ectomycorrhizosphere reads were performed with the analysis tools provided by MG-RAST, using the Greengenes (DeSantis et al. 2006), RDP (Cole et al. 2014) and SILVA SSU (Quast et al. 2012). As a reference database, we used a minimum cut-off identity of 60% and e-value of 5. The final taxonomy was decided, based on the best BLAST match for given representative sequence reads of the above dataset. The metagenome data are available at the NCBI - Sequence Read Archive with accession number: SRX8009931.

## Results

After QC and deduplication, a total of 50,213 reads with size 13,969,056 bp with an average length of 278 bp and G+C% 50±4 dataset were recovered from the active zone of ectomycorrhizosphere (RUGA-1). Taxonomic assignment was performed with the analysis tools provided by MG-RAST, using the SILVA SSU as a reference database with the default parameters, as it provides maximum number of matched reads, based on the BLAST score compared with RDP and Greengenes databases (Table 1). High species richness was observed in ectomycorrhizosphere. The reads were clustered using > 97% sequence identity to 1,905 microbial OTUs, which were classified and represented 24 bacterial and one archaeal phyla.

**Figure 2.**
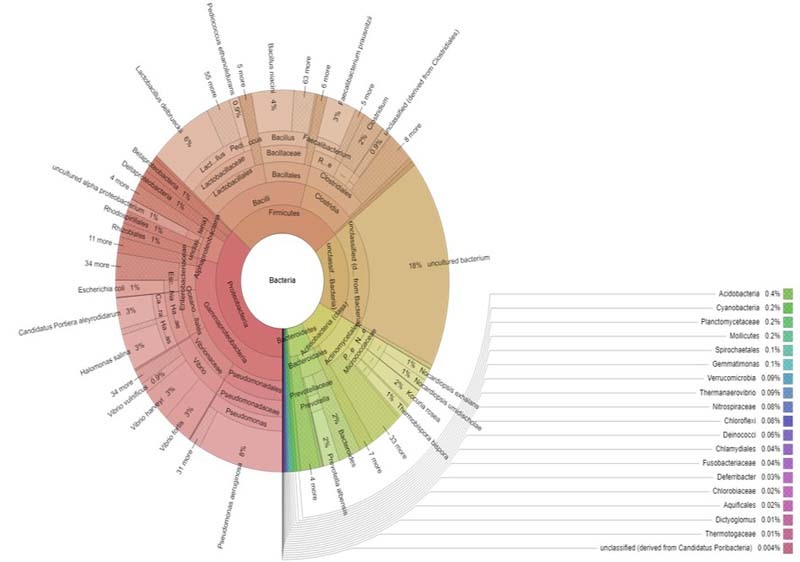
Krona chart of taxonomic affiliation of ectomycorrhizosphere and their relative abundance. The inner circle represents the higher taxonomic rank, while the outer circle represents a lower taxonomic rank up to the species level.

**Figure 3.**
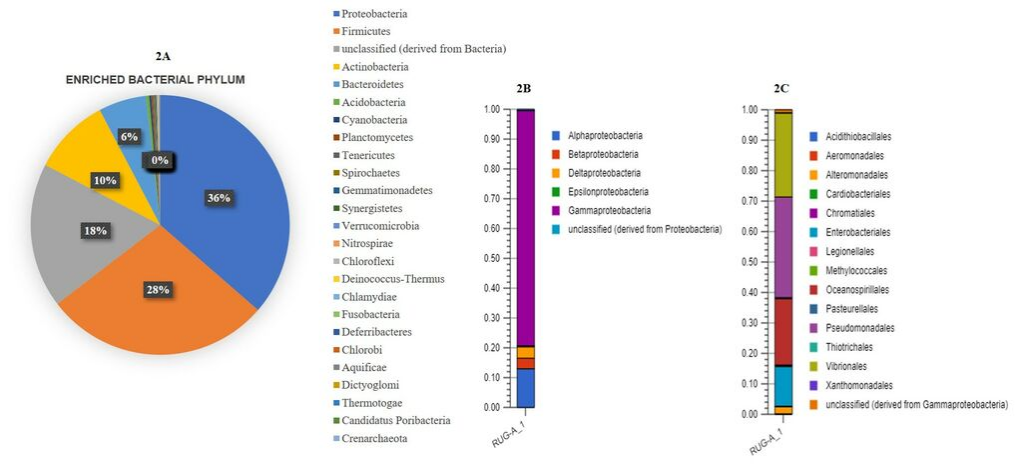
Taxonomic coverage of the ectomycorrhizosphere of *Astraeus*. **A.** Pie-chart showing the distribution of enriched microbial phyla; **B.** Stacked bar showing the distribution of relative abundant classes of Proteobacteria; **C.** Stacked bar showing of distribution of most relative abundant families of Gammaproteobacteria.

Bacteria were further classified into 45 classes, 103 orders and 224 families (Fig. 2). The most abundant phyla were Proteobacteria (36%), followed by Firmicutes (28%), Actinobacteria (10%) and Bacteroidetes (6%) (Fig. 3). In the active ectomycorrhizosphere zone, the most abundant superphylum Proteobacteria comprised of Gammaproteobacteria (28%), Alphaproteobacteria (4.5%), Deltaproteobacteria (1.3%), Betaproteobacteria (1.29%) and Epsilonproteobacteria (0.1%) (Fig. 3B), whereas Acidobacteria, Planctomycetes, Tenericutes and Spirochaetes were significantly more frequent.

Few members of Cyanobacteria and Archaea (Crenarchaeota) and pathogenic bacteria, such as *Escherichia, Salmonella, Vibrio, Helicobacter, Klebsiella* and *Shigella* were also identified in the study. A higher number of genera were identified in the active zone of ectomycorrhizosphere (n = 652). The most abundant species from the ectomycorrhizosphere was the Gram-negative rod-shaped *Pseudomonas aeruginosa* (8.2%) of Gammaproteobacteria, followed by the Gram-positive rod-shaped *Lactobacillus delbrueckii* (5%) of Firmicutes (Fig. 2). A number of significant reads fell under uncultured/unclassified OTUs (Suppl. material 1).

## Discussion

To our knowledge, this is the first high-resolution study of microbial diversity and their distribution in the active zone of the ectomycorrhizosphere of wild edible mushrooms of *Astraeus* from a dry deciduous forest of Shorea found in the red and sandy laterite soil with pH ranges from 5.0 to 6.0. In this study, we explored the abundance of microorganisms around the ectomycorrhizosphere and the hidden uncultured microbial communities. Such studies serve as baseline information for future research on the dynamics and distribution of microbial communities and how they relate to the physical environment and resilience. Mogge et al. (2000) and Khetmalas et al. (2002) reported that bacteria are common inhabitants in the mycorrhizosphere and they are more abundant in the mycorrhizosphere as compared to the bulk soil.

The most abundant class was methanotrophs of Gammaproteobacteria of phylum Proteobacteria (Fig. 3B and C). They play a vital role in phosphorus and iron mobilisation and are believed to be involved in mineral weathering and in plant- nutrition control as demonstrated by Köberl et al. (2017). Gammaproteobacteria contain members of the bacterial family, which are both medically and ecologically important. Pseudomonadaceae, Vibrionaceae, Halomonadaceae and Enterobacteriaceae were the most abundant families of Gammaproteobacteria (Fig. 3C). *Pseudomonas aeruginosa* is Gram-negative Gammaproteobacteria, rod-shaped, asporogenous, aerobic, opportunistic pathogen, which degrades polycyclic aromatic hydrocarbons and participates in biofilm formation and quorum sensing pathways (Botzenhart and Döring 1993). Firmicutes were very widely spread and abundant in the ectomycorrhizosphere soil. *Lactobacillus delbrueckii* is a Gram-positive, rod-shaped plant growth-promoting Firmicute that is active in the utilisation of recalcitrant carbon and inorganic nutrients (Llado et al. (2017).

Ecological processes of forest communities, though, are associated with microbes, yet, bacterial communities inhabiting the rhizosphere in forests have not been explored vis-a-vis grassland or agricultural systems. Several phyla and classes in this study have been found to dominate ectomycorrhizosphere bacterial communities. The comparative analysis of the data reveals similar bacterial community structure as reported in previous metagenomic assemblies from the ectomycorrhizosphere of oak forest (Uroz et al. 2012) and boreal forest (Pent et al. 2017), except for the abundance of *Pseudomonas.* The soils of ectomycorrhizosphere are rich in phosphate-solubilising bacteria, primarily of *Bacillus*, *Arthrobacterium*, *Agrobacterium*, *Micrococcus*, *Enterobacterium*, *Vibrio*, *Serbia*, *Rhizobium*, *Aeromonas*, *Burkholderia* and *Pseudomonas* (Liu 2019). Burke et al. (2008) reported ectomycorrhizosphere metagenome from Douglas fir EcM root tips, where EcM was dominated by Alphaproteobacteria and Bacteroidetes. Kataoka et al. (2012) observed Sphingomonas and Acidobacterium as abundant taxa in the pine mushroom *Tricholoma* from a forest of *Picea abies*. Stursova et al. (2012) reported Betaproteobacteria, Bacteroidetes and Acidobacteria as an abundant phyla. Similarly, Uroz et al. (2012) have also reported an abundance of Acidobacteria, Actinobacteria and Bacteroidetes from an oak forest, while Sphingomonas and Alphaproteobacteria from boreal forests were recorded by Pent et al. (2017) as abundant. However, in our investigation, we observed the highest abundance of Gammaproteobacteria, followed by Firmicutes and Actinobacteria. Previous studies of the metagenome of ecomycorrhizosphere of different soil types such as sandy, clay, loamy and podzol showed that soil types have a strong effect on the shape and structure of the rhizospheric microbiome of various environments, which may explain the taxonomic variations between them.

## Conclusion

Thus, the soil metagenomic analyses of the ectomycorrhizosphere, associated with wild mushrooms of *Astraeus*, revealed a distinct and unique assemblage of methanotrophic Gammaproteobacteria. However, other prokaryotic affiliates with a high percentage of unassigned taxa indicate scarce knowledge in the diversity of ectomycorrhizospheric communities.

## Supplementary Material

48DEC557-933D-5226-A6EA-1190155C8455 10.3897/BDJ.9.e63086.suppl1 Supplementary material 1 RUGA-1 Data type Genomic Brief description Taxonomic profiling of ectomycorrhizosphere soil. File: oo_536655.xlsx https://binary.pensoft.net/file/536655 VV, SSM, GS and SL

## Figures and Tables

**Table 1.** Summary statistics table.

**Reads Count**	**Sequences**	**bp Count**	**Mean Seq. Length**	**Mean GC** %	**No. of hits- Greengenes**	**No. of hits -RDP**	**No. of hits - Silva SSU**
Pre-QC	59,358	18,009,487 bp	303 ± 191 bp	50 ± 4%	**15,943**	**24,505**	**28,806**
Post-QC	50,213	13,969,056 bp	278 ± 199 bp	50 ± 4%			
